# QAPA: a new method for the systematic analysis of alternative polyadenylation from RNA-seq data

**DOI:** 10.1186/s13059-018-1414-4

**Published:** 2018-03-28

**Authors:** Kevin C. H. Ha, Benjamin J. Blencowe, Quaid Morris

**Affiliations:** 1 Department of Molecular Genetics, University of Toronto, 1 King’s College Circle, Toronto, ON M5A 1A8 Canada; 2 Donnelly Centre for Cellular and Biomolecular Research, University of Toronto, 160 College Street, Toronto, ON M5S 3E1 Canada; 3 Department of Computer Science, University of Toronto, 10 King’s College Road, Toronto, ON M5S 3G4 Canada; 4 Vector Institute, 661 University Avenue, Toronto, ON M5G 1M1 Canada

**Keywords:** High-throughput RNA sequencing, Alternative polyadenylation, Machine learning

## Abstract

Alternative polyadenylation (APA) affects most mammalian genes. The genome-wide investigation of APA has been hampered by an inability to reliably profile it using conventional RNA-seq. We describe ‘Quantification of APA’ (QAPA), a method that infers APA from conventional RNA-seq data. QAPA is faster and more sensitive than other methods. Application of QAPA reveals discrete, temporally coordinated APA programs during neurogenesis and that there is little overlap between genes regulated by alternative splicing and those by APA. Modeling of these data uncovers an APA sequence code. QAPA thus enables the discovery and characterization of programs of regulated APA using conventional RNA-seq.

**Electronic supplementary material:**

The online version of this article (10.1186/s13059-018-1414-4) contains supplementary material, which is available to authorized users.

## Background

Alternative cleavage and polyadenylation (APA) of pre-mRNA results in the formation of multiple mRNA transcript isoforms with distinct 3′ untranslated regions (UTRs). Approximately 70% of mammalian protein-coding genes contain multiple polyadenylation (poly(A)) sites [1, 2]. Thus, APA, much like alternative pre-mRNA splicing (AS) [3, 4], contributes extensively to eukaryotic transcriptome diversity and complexity. APA can occur within introns, or within 3′ UTR sequences [5], and as such can affect the composition of both protein coding and noncoding sequences in genes. Changes in 3′ UTR sequence through APA can significantly impact the fate of mature mRNA through the loss or gain of 3′ UTR sequences that harbor *cis*-regulatory elements recognized by microRNAs (miRNAs) and/or RNA-binding proteins (RBPs), as well as by affecting RNA structure [6, 7]. Through these mechanisms, APA plays important roles in the control of mRNA stability, translation, and subcellular localization [5, 8, 9]. However, our understanding of the regulation of APA and how it impacts gene expression is far from complete.

The polyadenylation machinery responsible for recognition of poly(A) sites involves interactions between several *trans*-acting factors and *cis*-elements. The core 3′ processing factors include cleavage and polyadenylation specificity factor (CPSF), cleavage stimulation factor (CstF), and cleavage factors I and II (CFI and CFII) [10–12]. Transcription of the poly(A) site by RNA polymerase II results in the recruitment of the above complexes via recognition of two surrounding sequence motifs in the nascent RNA. The first is a hexamer poly(A) signal located 10–30 nucleotides (nt) upstream of the poly(A) site that is recognized by CPSF [10]. In eukaryotes, the canonical, highly conserved hexamer is AAUAAA; however, other non-canonical variants also exist [13, 14]. The second is a G/GU-rich region downstream of the poly(A) site that is recognized by CstF [15]. This complex then recruits CFI and CFII to cleave the RNA at the poly(A) site [16], followed by poly(A) tail synthesis by polyadenylate polymerase (PAP) [17].

To facilitate a deeper understanding of APA, methods for the genome-wide mapping of poly(A) sites have been developed that employ high-throughput, directed sequencing of the 3′ ends of mRNAs [2, 18–23]. While these methods have provided invaluable insight into the global landscape of APA, they have not yet been extensively utilized, and consequently the availability of such data is currently limited. In contrast, there is a near exponential expansion in the number of conventional (i.e., whole transcript), mRNA-enriched high-throughput RNA sequencing (RNA-seq) datasets. Previous studies have demonstrated the potential of using conventional RNA-seq to characterize APA [4, 24–27]. However, the precise mapping of poly(A) sites from RNA-seq data is challenging due to read coverage biases at the 3′ end of transcripts, and poor yields of non-templated poly(A) tail-containing reads that can be reliably mapped to poly(A) sites [24] (KCHH, BJB, and QM unpublished observations). Moreover, another challenge is resolving the ambiguity of reads mapping to overlapping transcript isoforms [8]. To address these challenges, we posited the profiling of APA using RNA-seq data may be greatly enhanced by combining a comprehensive set of poly(A) site annotations with computational methods for accurate estimates of steady-state 3′ UTR abundance [28].

Accordingly, in this study we describe a new method, Quantification of APA (QAPA), that employs estimates of alternative 3′ UTR expression in combination with a significantly expanded resource of annotated poly(A) sites to demarcate UTR sequences that are specifically affected by APA. Demonstrating the effectiveness of our approach, we show that QAPA estimates for APA correlate well with those obtained using 3′ sequencing data, and that QAPA is more sensitive, efficient, and often more specific than other recently described methods for measuring APA. Using QAPA, we have profiled and determined new global regulatory features of APA during neurogenesis from a time series of RNA-seq data from differentiation of mouse embryonic stem cells (ESCs) to glutamatergic neurons [29]. Consistent with previous findings [30–32], a large subset of transcripts display progressive 3′ UTR lengthening during differentiation. We further observe sets of genes with 3′ UTR shortening and also genes that display temporally separated waves of shortening and lengthening during neurogenesis. Importantly, we also find that these changes in inferred APA are detected in genes that do not significantly overlap those with substantial steady-state changes in mRNA expression, alternative splicing, and transcriptional start sites. To probe regulatory mechanisms governing APA, we use QAPA data to train a new model of poly(A) site usage during neurogenesis and identify *cis*-elements that are predictive of this process. Collectively, our results demonstrate that QAPA facilitates the reliable detection and characterization of landscapes of alternative mRNA 3′ end processing from conventional RNA-seq data. As such, we envisage that QAPA will enable a more comprehensive definition of the programs of genes regulated by APA, as well as associated regulatory mechanisms, by leveraging the wealth existing RNA-seq data.

## Results

### Detection of APA from whole transcript RNA-seq data

**Fig. 1:**
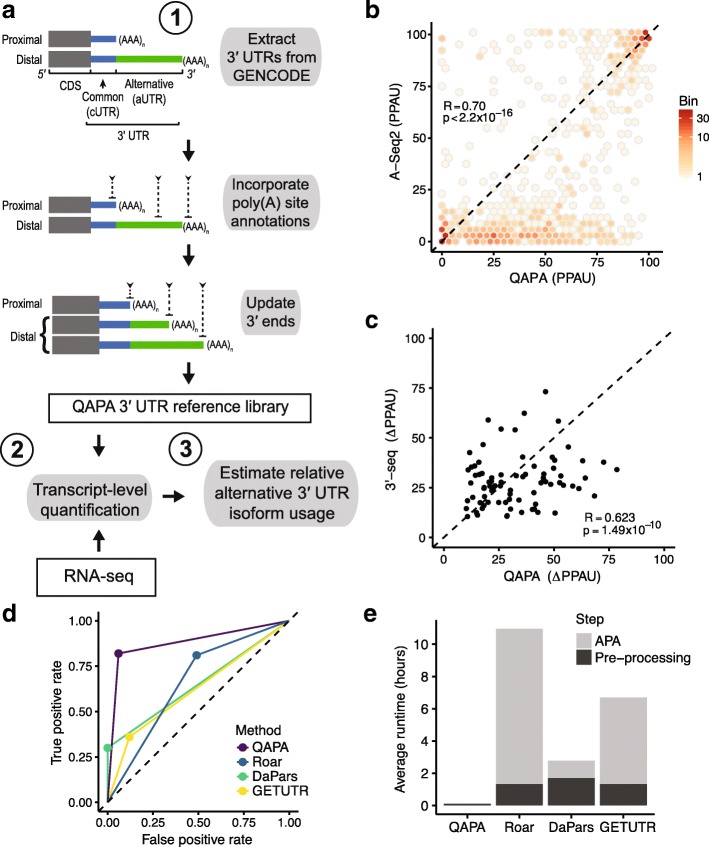
Profiling APA from RNA-seq. **a** Overview of annotated 3′ UTR library generation and QAPA method. *Top*: Terminal exons of two alternative 3′ UTR isoforms. The grey box indicates the coding sequence region. The *blue* region indicates the common region shared by both isoforms. The *green* region indicates the alternative region found only in the longer isoform. In (1), additional poly(A) site annotations (*inverted chevrons*) are used to refine the 3′ coordinates, as well as establish new isoforms. These new sequences are then used in (2) to measure expression from RNA-seq data and in (3) to estimate relative alternative 3′ UTR isoform abundance. **b** Hexbin scatterplot comparing PPAU estimates of 975 genes derived from HEK293 control samples assayed by RNA-seq (QAPA) [34] and A-seq2 [14]. Bins are colored by number of data points and the *dashed line* indicates the reference diagonal. **c** Scatterplot comparing ∆PPAU for 86 highly expressed genes between human skeletal muscle and brain tissue samples from RNA-seq (QAPA) [35] and 3′-seq [20]. **d** Receiver operating characteristic curves comparing performance of QAPA and other methods on simulated RNA-seq data. **e** Bar plot showing average runtime of each method on the same four RNA-seq samples divided into “pre-processing” stage for method-specific data preparation and “APA” stage for direct computation of APA results

QAPA quantifies APA levels using RNA-seq reads that uniquely map to 3′ UTR sequences demarcated by annotated poly(A) sites in last exons. The development and application of QAPA entailed establishing an expanded library of annotated poly(A) sites and 3′ UTR sequence. To this end, we constructed a reference library comprising sequences of last exons with distinct 3′ ends using GENCODE gene models for human and mouse [33] (Fig. 1a; see Additional file 1: Figure S1 and “Methods” for details). Many additional poly(A) sites detected by 3′-seq have not yet been incorporated into these or other existing gene models. As such, we expanded our library by including non-redundant annotations from two sources: PolyAsite database [14], a repository of poly(A) site coordinates from published 3′-end sequencing datasets, and the GENCODE PolyA annotation track [33], which contains manually annotated poly(A) sites. We used the compiled annotations (referred to below as “annotated poly(A) sites”) to update existing coordinates of proximal 3′ UTR sequences, and to establish coordinates for new instances of alternative 3′ UTR isoforms. In total, our set of annotated poly(A) sites represents 34,978 and 27,855 3′ UTR isoforms in human and mouse, respectively.

From analyzing our library, we observe that 74.3 and 65.7% of protein-coding genes contain two or more distinct poly(A) sites in human and mouse, respectively (Additional file 1: Figure S2), consistent with previous estimates [18, 20]. Because we incorporated only high confidence annotated poly(A) sites, i.e., those that are supported by multiple datasets (see “Methods”), our library may exclude potential poly(A) sites that have been previously reported. Hence, the numbers of protein-coding genes with multiple poly(A) sites in our library represent conservative estimates.

To quantify APA from the set of annotated 3′ UTR sequences with multiple APA sites, we applied Sailfish [28] to resolve reads that map to loci containing multiple transcript isoforms. We then inferred APA from differential expression of alternative 3′ UTR isoforms. We quantified APA using the metric “Poly(A) Usage” (PAU). The PAU for a 3′ UTR isoform is the ratio of its expression to the sum of the expression of all detected 3′ UTR isoforms from its gene. In this study, we focused on the PAU of the proximal 3′ UTR isoform (denoted as proximal PAU or PPAU), since APA is often regulated through the differential use of proximal poly(A) sites [20]. A lower value for PPAU thus implies that a distal poly(A) site is selected, and vice versa.

### Accuracy of QAPA estimates for alternative polyadenylation

To assess the performance of QAPA, we compared its PPAU estimates from conventional RNA-seq data to those computed from 3′-end sequencing data generated using two different protocols (A-seq2 [19] and 3′-seq [20]). For these analyses, we directly compared absolute PPAU and the change in PPAU (∆PPAU), as determined from each data type and method.

First, we used published RNA-seq and 3′-seq data from HEK293 cells [14, 34]. We estimated alternative 3′ UTR levels from the 3′-seq data by counting the number of A-seq2 reads mapping to each poly(A) site (see “Methods”), and computed PPAU as described above. Because these data were collected in different labs and from different stocks of HEK293 cells, and were generated using markedly different sequencing technologies, they exhibit a less than perfect correlation in overall steady-state mRNA expression profiles (*R* = 0.81, *p* < 2.2 × 10^–16^; data not shown). Despite these sources of variability, the QAPA PPAU estimates based on conventional RNA-seq data correlate well with those estimates determined using A-seq2 data (Pearson’s correlation *R* = 0.70, *p* < 2.2 × 10^−16^; Fig. 1b).

Next, to assess the accuracy of QAPA against a different 3′-end sequencing protocol (3′-seq [35]), and also in quantifying changes in APA, we compared ∆PPAU between human brain and skeletal muscle using RNA-seq data [35], with corresponding estimates from the same tissue types analyzed using 3′-seq data [20]. When considering APA events inferred by both methods in transcripts from genes with comparable expression between the two tissues (see “Methods”), the ∆PPAU values correlate well (Pearson’s correlation *R* = 0.62, *p* < 1.49 × 10^−10^; Fig. 1c). However, as in the case of the analysis of the HEK293 data described above, it is important to note that this degree of correlation represents an underestimate of the true correlation due to various sources of variability including—but not limited to—different sources of tissue samples, differences in overall gene expression profiles (“Methods”), and inherent differences in the sequencing methodologies.

### Comparison of methods for analyzing APA

We next compared the performance of QAPA with three other methods: Roar [26], DaPars [25], and GETUTR [27]. It is important to note in this regard that QAPA differs fundamentally from DaPars and GETUTR in its reference-based approach, and it also differs from all three methods by using fast, accurate pseudo-alignment techniques [28] to quantify 3′ UTR isoform levels. Roar does use a reference-based approach to identify APA changes; however, unlike QAPA its estimates for APA derive from counts of the number of reads in the extended alternative 3′ UTR (aUTR) region and in the common 3′ UTR (cUTR) region. In contrast, DaPars and GETUTR infer proximal poly(A) sites de novo by identifying significant changes in 3′ UTR read coverage.

To compare the four methods, we generated a synthetic RNA-seq dataset containing 200 multi-3′ UTR genes across two conditions, with three replicates per condition. Among these genes, 50 were assigned as 3′ UTR lengthening (∆PPAU > 20), 50 were assigned 3′ UTR shortening (∆PPAU < −20), and 100 served as no-change negative controls (−20 < ∆PPAU < 20). Overall, QAPA outperforms the other methods, as measured by the area under the receiver operating characteristic curve (AUC = 0.88; Fig. 1d); the AUC for Roar, DaPars, and GETUTR are 0.66, 0.65, and 0.62, respectively. In particular, DaPars and GETUTR detect fewer APA events (i.e., have a lower sensitivity) than reference-based approaches, suggesting that predicting proximal poly(A) sites de novo is relatively imprecise when using conventional RNA-seq. In this regard, utilizing a reference-based approach such as QAPA further provides a more comprehensive APA analysis from RNA-seq data. We also directly compared the performance of QAPA, Roar, DaPars, and GETUTR, in the detection of APA using the brain and skeletal muscle RNA-seq data described above. Consistent with the benchmarking results using simulated data, QAPA, followed by Roar, showed the highest degree of overlap of APA events that are also detected using 3′-seq from the same tissues (Additional file 1: Figure S3c).

Next, we measured the runtime that each of the four methods took to complete the analysis of four RNA-seq datasets [29], each of which comprised 20 million paired-end reads (see “Methods”). The total runtime was measured as the sum of two stages: (1) pre-processing steps required to prepare the data for APA analysis, including transcript abundance measurements and read alignment, and (2) inference of APA. Overall, because QAPA leverages the speed of alignment-free quantifications of transcript abundance, in contrast to conventional alignment procedures used by the other methods, it performed remarkably faster—i.e., less than 10 minutes compared to over 2 hours by the other methods (Fig. 1e; see “Methods” for details). Hence, QAPA provides an accurate, sensitive, and rapid reference-based approach for the quantitative profiling APA from RNA-seq data.

### Transcriptome-wide analysis of APA during neuronal differentiation

We next applied QAPA to investigate the genome-wide landscape of APA in the context of neuronal differentiation (ND), using conventional RNA-seq data generated from eight time points (with four replicates per time point) during differentiation of cortical glutamatergic neurons from embryonic stem cells (ESCs) [29]. We focused on a set of 3825 proximal 3′ UTR events measured with high confidence (see “Methods”) for downstream analyses (see Additional file 2 for a complete table of all events). To examine the reproducibility of QAPA quantification between biological replicates, we performed unsupervised hierarchical clustering on estimated PPAU values for each replicate. The results show that the replicates correlate well with each other (Additional file 1: Figure S4). Moreover, the samples clustered into three groups consistent with distinct developmental stages of ND defined in the original study [29]. Specifically, group 1 comprises days in vitro (DIV) −8 and −4, representing ESCs and neuroepithelial stem cells, respectively. Group 2 comprises DIV 0 and 1, representing radial glia and developing neurons, respectively. Finally, group 3 comprises DIV 7, 16, 21, and 28, representing successive stages of maturing neurons. These groupings mirror those derived from clustering the data based on gene expression profiles (data not shown), even though such changes involve a distinct subset of genes (see below). The clustering of PPAU profiles generated by QAPA thus reveals widespread changes in inferred APA regulation during ND.

**Fig. 2:**
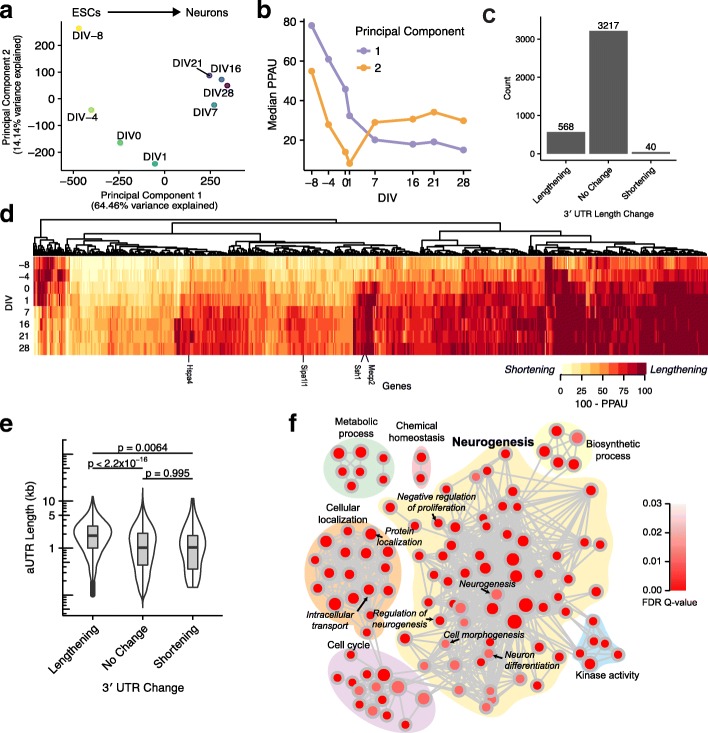
3′ UTRs lengthen during neuronal differentiation. **a** Scatterplot comparing the projections of QAPA PPAU profiles onto first (*x-axis*) and second (*y-axis*) principal components. Each point indicates the median values for a DIV stage over replicates. Mature neurons appear at DIV ≥ 7. Note that PC1 sorts samples by increasing development time as indicated above the plot. **b** *Lines* show the median PPAU (*y-axis*) of the top 100 3′ UTRs with largest absolute principal component loadings for PC1 (*purple*) and PC2 (*orange*) across increasing development time (*x-axis*). **c** Bar plot indicates the number of 3′ UTRs that lengthen (∆PPAU > 20), shorten (∆PPAU < −20), and do not change (|∆PPAU| ≤ 20) where ∆PPAU is defined as the difference in PPAU between ESC stages (DIV ≤ −4) and mature neuron stages (DIV ≥ 7). **d** Heat map displays PPAUs across DIV stages for the 608 genes whose |∆PPAU| > 20. *Columns* correspond to genes and are sorted to be consistent with the hierarchical clustering dendrogram shown above the heatmap. *Rows* correspond to DIV stages. To emphasize 3′ UTR lengthening, the distal PAU (= 100 − PPAU) is shown. **e** Combined violin and box plots comparing the lengths of the extended, alternative 3′ UTR (aUTR) regions in lengthening, shortening, and non-changing 3′ UTRs. *P* values were computed using Kolmogorov–Smirnov test. **f** Enrichment map summarizing gene set enrichment analysis results of Gene Ontology (GO) terms enriched in the genes with 3′ UTR lengthening. *Nodes* represent a GO term and *links* between two nodes indicate that more than 90% of the genes in the smaller term are also in the larger term

To elucidate the underlying patterns of APA changes during ND, we performed principal component analysis (PCA) on the PPAU values of each time point. We focused on the first two principal components (PCs), which described 64.5 and 14.1% of the data’s variance, respectively (Additional file 1: Figure S5a). PC1 captured APA changes consistent with a gradual lengthening (and, in rare cases, shortening) during ND (Fig. 2a; Additional file 1: Figure S5b, c). Moreover, by summarizing the PPAU profiles of genes with the highest weighting given by PC1, we observed that the transition to longer 3′ UTRs is more pronounced at early stages of ND (DIV 1) and is followed by a slower lengthening rate during neuronal maturation (Fig. 2b). Interestingly, in addition to these patterns, PC2 captures a pattern in which some 3′ UTRs lengthen as ESCs differentiate into glial cells, but subsequently shorten as they develop into neurons. To identify genes producing transcripts undergoing APA during ND, we calculated ∆PPAU between ESC and neuronal samples. Genes with ∆PPAU > 20 were deemed to have lengthening 3′ UTRs, while ∆PPAU < −20 were deemed to have shortening. By this definition, 568 (14.9%) and 40 (1.0%) genes lengthened and shortened, respectively, whereas 3217 did not display evidence of a change in UTR length (Fig. 2c, d). The strong bias toward lengthening is consistent with previous findings that 3′ UTRs often extend during neurogenesis [30–32, 36]. Our analysis expands the set of 3′ UTRs known to lengthen during this process, some of which are highlighted below.

To investigate differences in the properties of 3′ UTRs that lengthen, shorten, or don’t change, we compared the lengths of the longest aUTR region. Notably, the lengths of the aUTR regions in the lengthening group are significantly longer than those of the non-changing group (*p* < 2.2 × 10^−16^, two-sided Kolmogorov–Smirnov test), whereas the aUTR lengths of this latter group are not significantly different from those of the shortening group (Fig. 2e). This is in agreement with previous observations that genes with tissue-dependent 3′ UTR isoform expression tend to have longer 3′ UTR lengths compared to constitutively expressed isoforms [20]. Overall, the median lengths of aUTRs in lengthening, shortening, and non-changing 3′ UTRs are approximately 1.9, 1.4, and 1.0 kb, respectively.

We next performed gene set enrichment analysis (GSEA) [37] to assess whether genes associated with lengthening or shortening 3′ UTRs belong to common biological functions or pathways. No terms are significantly enriched in the set of genes with 3′ UTR shortening during ND, possibly due to the small size of this group. In contrast, multiple Gene Ontology (GO) terms associated with ND are enriched in genes with lengthening 3′ UTRs; these include neurogenesis, nervous system development, embryo development, cell morphogenesis, proliferation, and localization (Fig. 2f).

**Fig. 3:**
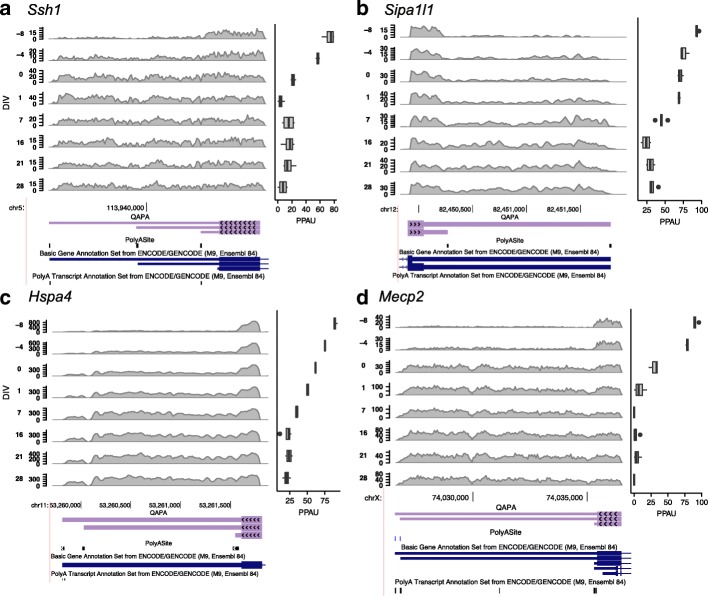
Examples of lengthening events detected by QAPA based on updated 3′ UTR isoform annotations. Four examples of 3′ UTR lengthening: **a** *Ssh1*, **b** *Sipa1l1*, **c** *Hspa4*, and **d** *Mecp2*. In each example, RNA-seq read coverage of each 3′ UTR at each DIV stage (*rows*) is displayed (using the first replicate of each stage as a representative example). A schematic from the UCSC Genome Browser (mm10) [82] for each 3′ UTR is shown below. Four annotation tracks are shown. From *top* to *bottom*, these tracks are: QAPA-annotated 3′ UTR models, PolyAsite [14] annotations with score ≥ 3, GENCODE [33] gene annotation models, and GENCODE Poly(A) track annotations (except for *Sipa1l1*, in which no supporting GENCODE Poly(A) data were found). *Ssh1*, *Sipal1l*, and *Mecp2* are shown in the reverse strand orientation. For *Mecp2*, although an intermediate GENCODE poly(A) site is present, there was insufficient support from PolyAsite annotations and thus it was not used to define a 3′ UTR model (see “Methods”). The horizontal boxplots to the *right* show the PPAU values across replicates in each corresponding DIV stage to the row

We identified new examples of genes that lengthen during neuronal differentiation as a consequence of applying QAPA in conjunction with our expanded library of poly(A) sites. Four examples are shown in Fig. 3, and additional cases are shown in Additional file 1: Figure S6. In the example of the gene *slingshot protein phosphatase 1* (*Ssh1*; Fig. 3a), the GENCODE gene model indicates a proximal 3′ UTR of 47 nt. In contrast, our analysis supports a longer proximal 3′ UTR of 557 nt, consistent with PolyAsite annotations, GENCODE Poly(A) track annotations, and visualization of RNA-seq read mappings. In the case of *signal induced proliferation associated 1 like 1* (*Sipa1l1*) and *heat shock 70 kDa protein 4* (*Hspa4*) (Fig. 3b, c), each gene is annotated by a single GENCODE 3′ UTR isoform whereas our library and RNA-seq data support two and three distinct 3′ UTR isoforms, respectively. Finally, we detected previously validated 3′ UTR lengthening in *methyl CpG binding protein 2* (*Mecp2)* [38], a gene causally linked to Rett Syndrome that is critical for normal brain development [39] (Fig. 3d). QAPA analysis in conjunction with the employment of our expanded 3′ UTR library thus can capture more isoforms than current annotation resources, as also supported by our benchmarking comparisons described above.

### Differential APA and steady-state gene expression changes during ND largely involve distinct subsets of genes

Given the large program of changes that occur during ND, including numerous changes in total steady-state mRNA abundance, we next investigated whether the observed 3′ UTR length changes during ND are primarily due to differential recognition of alternative poly(A) sites, or possible changes in the differential stability of the proximal and/or distal 3′ UTR isoforms that may affect steady-state expression levels of the corresponding isoforms. To address this question, we identified genes with overall differential steady-state mRNA expression levels (i.e., changes involving all isoforms from a gene) and genes in the same data that display QAPA-inferred differential APA during ND, and then asked whether there was a statistically significant overlap between these two sets of genes.

**Fig. 4:**
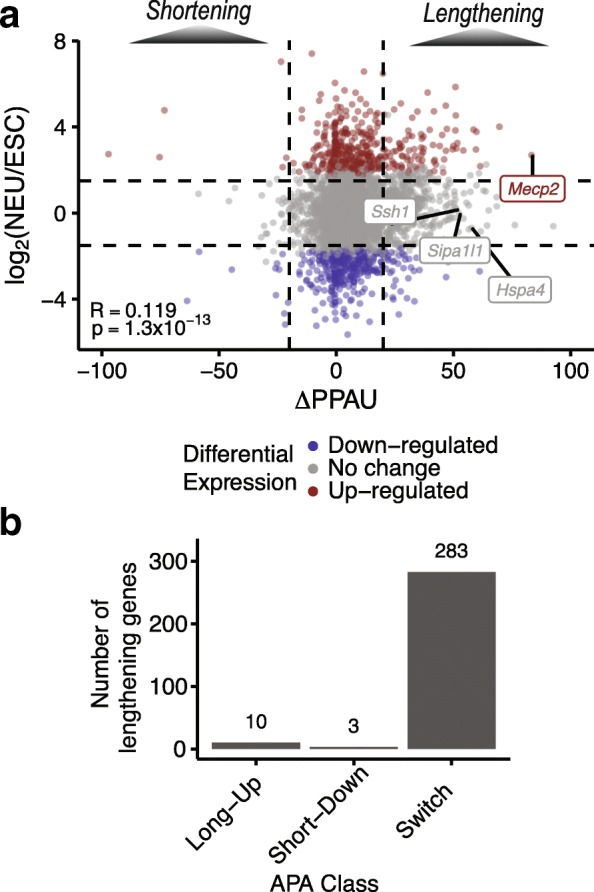
APA changes during ND are rarely correlated with steady-state mRNA expression changes. **a** Comparison between mRNA expression changes (*y-axis*) and APA changes (*x-axis*) for 3825 analyzed genes. Lengthening 3′ UTRs are indicated on the *right* (∆PPAU > 20), while shortening 3′ UTRs are on the *left* (∆PPAU < − 20). Genes with statistically significant differential up- or down-regulation are indicated by *red* and *blue dots*, respectively (|log_2_ fold change| > 1.5, FDR < 1%). Examples of lengthening 3′ UTRs from Fig. 3 are labeled. *Dotted horizontal lines* indicate log_2_ fold change thresholds, while *dotted vertical lines* indicate ∆PPAU thresholds. **b** Bar plot showing distribution of lengthening 3′ UTRs across classes based on isoform expression changes between proximal and distal 3′ UTRs: *Switch*, *Long-Up*, or *Short-Down*

To this end, we used DESeq2 [40] to identify genes that are differentially expressed between ESCs (DIV −8 and −4) and maturing neurons (DIV 7, 16, 21, and 28). Of 3825 analyzed genes, we observe that 423 (11.1%) display a significant increase in expression and 394 (10.3%) a decrease in expression during differentiation (Additional file 1: Figure S7a; |log_2_ fold change| > 1.5, FDR < 0.01, where fold change is the ratio between neuronal expression and ESC expression). Notably, among a total set of 608 genes with QAPA-inferred lengthening or shortening 3′ UTRs, the large majority (460, 75.7%) do not overlap those genes with significant expression changes (Table 1). Moreover, this subset also did not display significant changes in mRNA expression when comparing ESCs with an earlier stage of ND (DIV 1; Additional file 1: Figure S7b). However, of the 568 genes with 3′ UTR lengthening, 88 (15.5%) display increased steady-state mRNA expression, and 44 (7.8%) show decreased expression (Fig. 4a). By independently comparing the number of lengthening and shortening genes with differential expression changes to those genes without associated expression changes, we observed a higher than expected overlap between genes with both 3′ UTR lengthening and increased expression, and a barely significant overlap between 3′ UTR shortening and decreased expression (*p* = 0.002 and *p* = 0.02, two-sided Fisher’s exact test, Bonferroni correction).

**Table 1:** Summary of genes with QAPA-inferred APA changes and significant differential steady-state mRNA expression changes measured by DESeq2 [40] (|log_2_ fold change| > 1.5 and FDR < 0.01)

		Differential expression		
		Increase	No change	Decrease
APA	Lengthening	88	436	44
	No change	329	2548	340
	Shortening	6	24	10

We next investigated the extent to which QAPA-detected 3′ UTR changes during ND are represented by genes for which there are changes in the steady-state expression of only one of the resulting proximal (short) or distal (long) isoforms, versus genes for which there are reciprocal changes in levels of these isoforms. For this analysis, DEXSeq [41] was used to detect significant changes in the expression of the proximal or distal 3′ UTR isoforms, particularly focusing on lengthening genes. We classified these genes as *Long-Up* if only the distal isoform is up-regulated during ND, *Short-Down* if only the proximal isoform is down-regulated, and *Switch* if the distal isoform is up-regulated and proximal isoform is down-regulated. Overall, a total of 296/568 (52.1%) genes with 3′ UTR lengthening could be confidently assigned to one of these three classes (Fig. 4d). Importantly, the *Switch* class represents the majority (283) of events, whereas the *Long-Up* and *Short-Down* classes represent only ten and three genes, respectively (examples in Additional file 1: Figure S8). These results are thus further consistent with our observation that the large majority of genes with changes in steady-state gene expression levels during ND do not overlap those genes with QAPA-inferred APA. Moreover, the results suggest that the majority of the inferred APA events that involve reciprocal changes in proximal and distal isoform expression likely arise from differential APA regulation. In the case of the smaller groups of genes that are either specifically long- or short-regulated, it is probable that additional post-transcriptional mechanisms, including miRNA- and RBP-mediated regulation of transcript stability, result in unidirectional changes that affect the relative ratios of these isoforms.

### Differential APA, alternative splicing, and transcription start site selection are largely independent regulatory events during neuronal differentiation

**Fig. 5:**
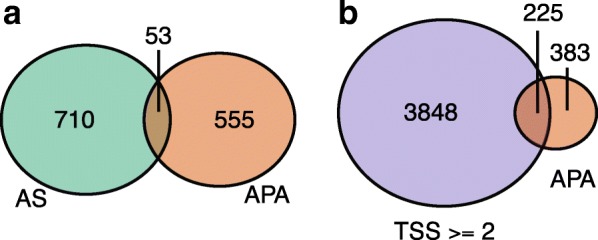
APA during neuronal differentiation is generally independent of alternative splicing and multiple transcription start sites. **a** Venn diagram showing the overlap between 3′ UTR lengthening and shortening genes (*right*) and genes with differentially regulated AS events [50] (*left*). **b** Venn diagram showing the overlap between 3′ UTR lengthening and shortening genes (*right*) and genes with more than one distinct transcription start site (*left*). Neither overlap is statistically significant (*p* = 0.56 and 0.49, respectively, Fisher’s exact test)

Previous studies have demonstrated links between splicing and APA. For example, specific splicing regulators such as SRRM1 [42] and NOVA [43] control 3′-end formation, and components of the cleavage polyadenylation machinery can influence splicing [44–46]. Another example is the spliceosome factor U1 small nuclear ribonucleoprotein regulating the usage of cryptic intronic poly(A) sites [47, 48]. Moreover, selection of alternative last exons is coupled with APA in the same exons [49]. However, overall, it is not clear to what extent APA (occurring within the 3′ UTR) and AS changes (independent of terminal exon selection) act independently or in a coordinated fashion to impact gene regulation. To address this in the context of ND, we investigated whether genes with differential APA significantly overlap those with differentially regulated AS events. We carried an analysis of AS on the same dataset (see “Methods”) that detected cassette exons (including microexons of length 3–27 nt) and alternative 5′/3′ splice sites. Only 53/608 (8.7%) of genes with QAPA-inferred APA contain one or more differentially regulated AS events (Fig. 5a). However, this overlap is not significantly different from the overlap between genes with no inferred APA changes and those with neural-regulated AS (*p* = 0.56, two-sided Fisher’s exact test). We also compared genes with QAPA-detected APA with an independently defined set of genes with neural-regulated AS events [50] and, again, did not observe any significant overlap (*p* = 0.37, two-sided Fisher’s exact test; Additional file 1: Figure S9a).

Since APA has previously been linked to changes in transcription initiation [51], we additionally asked whether genes with QAPA-inferred APA are enriched for multiple transcription start sites. We observe that 259/608 (42.6%) such genes contained two or more distinct start sites (Fig. 5b, Additional file 1: Figure S9b). However, again, this overlap is not significantly different from that overlap with genes lacking APA (*p* = 0.49, two-sided Fisher’s exact test).

Taken together, these results provide evidence that APA is a distinct layer of regulation that is largely independent of programs of differential gene expression, AS, and transcription start site selection, during ND. Nevertheless, it is important to bear in mind that in specific cases these processes are coupled and can influence each other [45, 46].

### Modeling the APA regulatory code using QAPA data

Because APA appears to act largely independently of other regulatory mechanisms, and because a parsimonious explanation for our observations is that APA changes are largely regulated by differential choice of poly(A) sites, we assembled models for inferring the role of *cis*-elements that control proximal poly(A) site choice. In this regard, the full set of *cis*-regulatory instructions for the regulation of APA is not known. Moreover, QAPA, coupled with our expanded resource of annotated poly(A) sites and UTR sequences, provides a considerable increase in quantitative estimates for inferred APA available for modeling, and therefore has the potential to afford a greater resolution in inferring an APA code. To investigate this possibility, we used QAPA predictions generated from the analyses described above to quantitatively model poly(A) site usage in the context of ND. We trained our model to predict PPAU levels using QAPA estimates from the ND RNA-seq data [29] described above and then inferred *cis*-elements (and potential cognate *trans*-factors) controlling choice of poly(A) sites.

Using an approach similar to that applied previously to predict regulated alternative splicing [52], we first collected and analyzed a variety of features within 300 nt upstream and 300 nt downstream of each poly(A) site. The features were assigned to four broad groups: sequence content, polyadenylation-related, RBP motifs, and conservation. The first group included features describing dinucleotide sequence content. The second included features indicating the presence or absence of 18 possible poly(A) signals within 50 nt upstream of the poly(A) site, as well as the enhancer element UGUA. Among the 18 poly(A) signals, 12 were initially defined by Beaudoing et al. [13], and an additional six were defined by Gruber et al. [14]. We also included features describing the dinucleotide at the polyadenylation site. The third group contained features representing 204 experimentally defined RBP motifs from RNAcompete [53]. Each RBP motif was also scored for its computationally predicted accessibility [54] (see “Methods” for details). Scores were summed within 100-nt bins between 300 nt upstream of a proximal poly(A) site to 300 nt downstream, resulting in six binned features per motif for a total of 1224 motif features. Finally, we also included features describing the conservation profile upstream and downstream of the poly(A) site. In total, we collected 1296 features (Additional file 3). We built a regression model that describes the propensity or “site strength” of a poly(A) site using the features described above, as poly(A) site strength is thought to be due to a combination of many factors [55]. Using the ND RNA-seq dataset [29], we computed the mean PPAU value across all samples for each gene. Constitutively expressed proximal poly(A) sites will have a high mean PPAU, while differentially regulated poly(A) sites will have low to mid-range mean PPAU. For this model, we included proximal poly(A) sites to reflect APA, as well as single, constitutively expressed poly(A) sites (i.e., genes with a single site), which have a PPAU value of 100. In the latter case, we assume these to be examples of strong poly(A) sites, and that the mechanisms for processing a single site are not necessarily different from those of a proximal site.

**Fig. 6:**
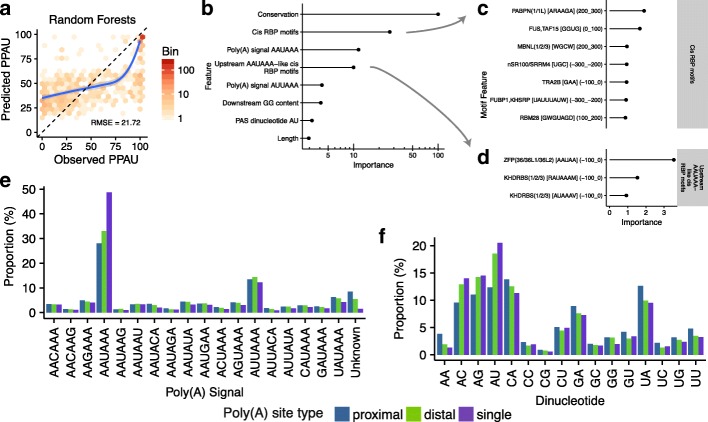
Modeling the APA regulatory code using random forests. **a** Hexbin scatterplot comparing PPAU predictions made by random forests model on genes in the ND RNA-seq dataset [29] to the observed QAPA-assigned PPAU values. Only data on held-out genes not used in the training the model are shown here. Higher values indicate increased usage and vice versa. Bins are colored by number of data points. The *dashed line* indicates the reference diagonal. The *blue line* represents a polynomial spline of best fit to the data. **b** Dot plot showing the top six features from the model. The *x-axis* indicates the importance of each feature (see “Methods”), scaled between 0 and 100. Higher values indicate that the feature has stronger predictive value than lower values. Note that the *Conservation*, *Cis RBP motifs*, and *Upstream AAUAAA-like cis RBP motifs* features shown are the sum of the importances from all the corresponding binned conservation-related and motif-related features. **c** Zoom-in dot plot showing the importances of the top eight motif features from the Cis RBP motifs set. This set consists of RBP motifs that are not similar to the AAUAAA poly(A) signal. Each motif is labeled according to the corresponding RBP, IUPAC motif, and bin region. **d** Zoom-in dot plot showing the importances of individual Upstream AAUAA-like RBP motifs. These features are likely predictive due to their similarity to the canonical poly(A) signal AAUAAA. **e** Distribution of 18 poly(A) signals in mouse, grouped by poly(A) site type: proximal (poly(A) site closest to stop codon), distal, and single (genes with one poly(A) site). **f** Similar to **e**, distribution of 16 poly(A) site dinucleotides, grouped by poly(A) site type

To train our model, we compared three algorithms: linear regression with LASSO regularization [56], random forests [57], and gradient tree boosting [58]. These algorithms were chosen for their ability to carry out feature selection. Reducing the number of features in this manner thus provides interpretable insight into *cis*-elements that are most important for prediction of poly(A) site selection. A model was trained for each method using cross-validation, and evaluation was carried out on held-out test data (see “Methods”). Overall, random forests and gradient tree boosting outperformed LASSO (root-mean-square error (RMSE) = 21.72, 21.87, and 26.48, respectively; Fig. 6a for random forests and Additional file 1: Figure S10 for LASSO and gradient tree boosting). Furthermore, all three methods outperformed a baseline model that predicts only the mean PPAU from the training data (RMSE = 37.46), suggesting that our models contained features that are predictive of PPAU.

We next investigated the importance of features in the random forests model (Fig. 6b–d). Among the top features, conservation surrounding the proximal poly(A) site is strongly associated with site strength as well as the two poly(A) signals, AAUAAA and AUUAAA, the poly(A) site dinucleotide AU, and downstream GG dinucleotide content. To determine the prevalence of the latter feature groups, we examined the distribution of all 18 poly(A) signals and 16 poly(A) site dinucleotides in the poly(A) sites of proximal, constitutive, as well as distal 3′ UTRs. As expected, the signals AAUAAA and AUUAAA were the two most frequent elements in all three types (Fig. 6e). Among the AAUAAA-containing events, constitutive 3′ UTRs are the most prevalent, followed by distal and proximal 3′ UTRs. This is in agreement with previous reports suggesting that proximal poly(A) sites are typically less often selected and thus are less likely to contain a strong poly(A) signal [55]. The poly(A) site dinucleotide AU was the most frequently observed poly(A) site for single and distal poly(A) sites, while CA was the most frequent in proximal poly(A) sites (Fig. 6f). Similarly, we observed that the downstream content of GG (measured in the 300-nt region downstream of the poly(A) site) provided some predictive value. Finally, several RBP motifs also collectively provided substantial predictive value. As several of the RBP motifs closely resembled the canonical poly(A) signal AAUAAA, we separated the motif features as either upstream AAUAA-like, located within the (−100, 0) bin (Fig. 6c), and non-AAUAAA-like (Fig. 6d). The upstream AAUAAA-like features are among the top scoring motifs and likely overlap the poly(A) signal features. The other non-AAUAAA-like features individually provided a much smaller amount of predictive value. This suggests that while collectively RBP motifs provide considerable predictive value in site strength, their involvement is complex and individual RBPs each contribute to APA regulation with small effect sizes and in different contexts. In summary, our model highlights various sequence features that are important for the overall prediction of proximal poly(A) site usage and further indicates that, in contrast to the code underlying tissue-dependent regulation of AS, does not comprise RBP motif *cis*-features that act widely to control APA.

## Discussion

In this study, we present a new computational approach, QAPA, to quantitatively infer APA from conventional RNA-seq data, by profiling 3′ UTR isoforms demarcated by annotated poly(A) sites. Facilitating the application of this method, we have introduced a more comprehensive resource of annotated poly(A) sites and alternative 3′ UTR sequences for human and mouse that significantly improves on existing gene model annotations. To resolve overlapping isoforms, our method employs a recent transcript-level quantification strategy based on k-mer frequencies [28], which obviates the compute-intensive and time-consuming steps of alignment of reads to a reference genome or transcriptome. Using these combined approaches, QAPA directly estimates absolute alternative 3′ UTR isoform expression and then computes the relative expression of each isoform among all isoforms to assess APA. When developing QAPA, we tested incorporation of information from chimeric reads containing non-templated poly(A) stretches to locate poly(A) sites [24]. However, we found this approach to be unreliable due to very low yields of such reads, and the poor quality of the templated portion of the reads, and as such including these reads did not enhance performance (data not shown).

We show that QAPA estimates for APA correlate well with those derived from 3′-end sequencing methods, despite inherent sources of variability due to technical differences in sequencing methods, where the samples were sequenced, and expression levels between the samples. A major goal of this study was to introduce a reliable method for inferring APA when 3′-end sequencing data are unavailable. In this regard, currently there is a limited amount of such data compared to conventional RNA-seq data. However, we support continued generation of 3′-end sequencing data, as it represents an effective approach for the definition of poly(A) sites and the characterization of APA regulation. In addition to displaying comparable accuracy as 3′-end sequencing data in inferring APA, in benchmarking comparisons we observe that QAPA has an overall greater sensitivity and speed than other recently described methods [25–27] for inference of APA from RNA-seq data. Finally, by performing QAPA analysis of conventional RNA-seq data from a time course of ND from ESCs [29], we provide an extensive resource of quantitative estimates of APA during ND and further use these data to model an APA regulatory code. These results thus demonstrate the potential of QAPA for greatly expanding our knowledge of APA by harnessing the wealth of existing conventional RNA-seq data.

A limitation of QAPA is that it requires poly(A) sites to be pre-defined. In the present study, this issue is mitigated by the generation of a greatly expanded resource of annotated poly(A) sites that incorporates data from 3′-seq and other resources. Moreover, the addition of future poly(A) site data (e.g., from new 3′-end sequencing data) to this resource will further increase the power of QAPA. It should be noted that the de novo discovery of APA from conventional RNA-seq data is challenging, given the uneven distribution of reads across 3′ UTR sequence. Hence, coupling a comprehensive annotation of experimentally supported poly(A) sites is therefore a critical component of QAPA’s inference of poly(A) site selection from conventional RNA-seq data.

Using QAPA to analyze APA in longitudinal RNA-seq data from glutamatergic ND confirms previous reports that 3′ UTR lengthening is the predominant APA pattern during differentiation [30–32, 36], with smaller subsets of genes displaying shortening or successive waves of lengthening and shortening, or vice versa. This analysis further defined new cases of inferred APA, overall progressive lengthening as ESCs differentiate into neural precursor cells, and the observation that genes that undergo 3′ UTR lengthening overall have a longer median 3′ UTR length (1.9 versus 1.4 kb) compared to those genes that do not undergo lengthening, thus affording greater potential for miRNA-, RBP-, or RNA structure-based regulation [9, 32, 38]. Furthermore, the majority of inferred APA events are not associated with significant and selective changes in steady-state 3′ UTR isoform levels during ND. While this is consistent with previous observations that genes subject to regulation by APA largely do not overlap with genes with differential expression in the same biological context [19, 31, 59], we do observe a higher than expected number of genes with 3′ UTR lengthening that display accompanying increased expression during ND. Hence, possible coupling of APA with steady-state mRNA expression changes impacts a relatively small number of genes and may arise through mechanisms involving miRNA- and RBP-mediated control of mRNA turnover. One such example is *Mecp2*, in which its long 3′ UTR isoform has been shown to be post-transcriptionally regulated by a coordinated program of miRNAs and RBPs during ND [38]. Furthermore, among the genes with inferred APA during ND, we do not observe significant overlap with genes that contain (non-terminal exon) neural-regulated AS and multiple transcription start sites.

To investigate the regulatory code governing APA, we developed models to predict poly(A) site usage. Previously, classification models have been used to predict functional poly(A) sites in genomic sequence [60–62], as well as tissue-specific poly(A) sites from constitutive poly(A) sites [63, 64]. Here, our regression models employ a set of features that represent sequence properties flanking each poly(A) site to predict usage. We trained the models using LASSO, random forests, and gradient tree boosting. Overall, our best models were achieved by the latter two, both of which outperformed a baseline model that predicts the average PPAU across the ND samples. Features that contributed the most predictive power are conservation, the poly(A) signals AAUAAA and AAUAAA, and to a smaller extent poly(A) site dinucleotide AU. The conservation patterns surrounding the poly(A) site are in part due to conserved poly(A) signals and downstream elements [20]. In the case of poly(A) site dinucleotides, while CA has been reported as the preferred poly(A) site dinucleotide [65], a subsequent study revealed a nucleotide preference order of A > U > C ≫ G at the cleavage site [66]. We observed that AU is the most frequent dinucleotide (Fig. 5d); however, our model suggests that AU weakly predicts poly(A) site selection. We also detect relatively small contributions of specific RBP motifs to overall poly(A) site usage, likely because individual RBPs control only small subsets of target events and in specific contexts. These results thus highlight the inherent challenge of *in silico* inference of an APA code that accounts for regulatory behavior in different biological contexts. We propose that the application of QAPA to the enormous wealth of existing conventional RNA-seq data may provide sufficient genome-wide measurements of poly(A) site usage to significantly enhance further efforts directed at inferring the APA code. Based on our observations in the present study, we expect that such an expanded analysis will define relatively small sub-networks of APA events controlled by individual RBPs or other regulatory factors.

## Conclusions

In this study, we developed and applied QAPA, a new method that uses conventional RNA-seq data to infer poly(A) site selection and alternative 3′ UTR usage. We further introduced a greatly expanded resource of poly(A) site annotations that are used by QAPA to infer APA. As exemplified by its application to a time series of ND RNA-seq data, QAPA facilitates the systematic discovery and characterization of APA across diverse physiologically normal and disease conditions. Also, as demonstrated in the present study, such expanded datasets for poly(A) site selection generated by QAPA facilitate modeling of the APA code.

## Methods

### Curating a library of 3′ UTR isoform sequences

We used gene models based on the GENCODE [33] basic gene annotation set version 19 and M9 for humans (hg19) and mouse (mm10), respectively, to build our database of 3′ UTRs from protein-coding genes. First, we perform filtering on these gene models to identify 3′ UTR isoforms that are likely to be part of stable mRNA transcripts. Then we used additional poly(A) site annotation sources to refine the 3′ end of some of the 3′ UTR isoforms, or to add new isoforms where additional poly(A) sites appear that are not present in the GENCODE basic annotations. See Additional file 1: Figure S1 for a flow chart of the procedure. We performed a series of filtering steps to pre-process the 3′ UTR isoforms. First, we removed 3′ UTRs with introns that are likely to lead to nonsense-mediated decay and 3′ UTRs that are not at the 3′-most end of the coding region. We identified the latter by removing 3′ UTRs that overlap with the coding region or introns. Then, we extracted the genomic coordinates of terminal exons from each transcript, which include both the 3′ UTR and the adjacent coding sequence region (Fig. 1). Note that our filtering ensures that all these terminal exons have the same 5′ start site. For convenience and clarity, we refer to these terminal exons as 3′ UTRs. Finally, we excluded 3′ UTRs shorter than 100 nt in length, which are difficult to quantify.

Next, we used two additional poly(A) site annotation sources to refine the 3′ ends of our set of 3′ UTRs and to generate new 3′ UTR isoforms where a well-supported poly(A) site appeared within an existing 3′ UTR. These annotation sources were the GENCODE basic poly(A) annotation track [33], and the PolyAsite database (http://polyasite.unibas.ch/; accessed on December 2016) [14]. Specifically, we included all GENCODE entries and only PolyAsite entries that had three or more supporting 3′-end sequencing datasets (score ≥ 3) and were labeled as “TE” or “DS” (for downstream poly(A) sites). Collectively, we will refer to a poly(A) site from one of these sources as an annotated poly(A) site. We used the annotated poly(A) sites in two ways: to refine the 3′ end of nearby 3′ UTRs, or to generate new 3′ UTR isoforms. Note we used annotated poly(A) sites from GENCODE only to refine the 3′-ends of nearby 3′ UTR; sites from PolyAsite were also used to generate new 3′ UTR isoforms.

To update 3′ ends of 3′ UTRs, thereby accounting for slight variability in precise cleavage sites, if an annotated poly(A) site was located within 24 nt of the existing 3′ end coordinate of a 3′ UTR, then we replaced its coordinate with that of the annotated poly(A) site. The 24-nt cutoff is based on previous poly(A) site clustering pipelines [1]. We generate a new 3′ UTR isoform if an annotated poly(A) site otherwise occurs within an existing 3′ UTR and the annotated poly(A) site source is from PolyAsite and is supported by four or more 3′-seq datasets (note this is a more stringent criteria than we use for allowing a PolyAsite to update a 3′ end). This new 3′ UTR isoform is assigned the same 5′ end as all the other 3′ UTR isoforms for that gene. Finally, we perform a final merge of 3′ UTRs with 3′ ends within 24 nt of each other to produce a non-redundant set of isoforms. All genomic interval operations were performed using pybedtools [67]. Sequences were extracted using bedtools getfasta [68].

### Data processing of RNA-seq datasets

Transcript-level expression of 3′ UTRs was measured using Sailfish v0.8.0 [28] and our curated reference library of 3′ UTR sequences. To quantify the relative usage of 3′ UTR isoforms (and thus differential poly(A) site usage), we calculate the relative expression of a 3′ UTR over the total expression level of all 3′ UTRs in a gene, defined by a metric called Poly(A) Usage (PAU):

$$ {PAU}_{ig}=\frac{e_{ig}}{\sum \limits_j{e}_{jg}}\ast 100 $$

where *g* is a given gene, *e*_*ig*_ is the expression level of isoform *i* in *g*, measured in transcripts per million (TPM). RNA-seq read coverage was visualized using the R package Gviz [69].

### Data processing of 3′-end sequencing datasets

For A-seq2, reads were processed as described in Gruber et al. [14], with some modifications. Briefly, after removing adapters, reads were reverse complemented, collapsed using FASTX-Toolkit, and aligned to the human reference genome (hg19) using Bowtie2 v2.2.6 [70] with --local option. Next, we used filtering criteria outlined in Gruber et al. [14] and further filtered the alignments to remove non-uniquely mapping reads (MAPQ < 10), reads with more than two Ns, reads with more than 80% adenines, and reads where the last nucleotide is adenine. To annotate and quantify poly(A) sites, reads overlapping the PolyAsite (hg19) database were quantified using bedtools intersect (with options –s, −wa, and –c) [68], forming poly(A) site clusters. To ensure that all reads that mapped near a poly(A) site cluster were counted, we extended clusters less than 30 nt in length by 15 nt on either side. An equivalent PAU metric was used to quantify the relative usage of poly(A) sites as described above. In this case, the relative proportion of read counts at a given poly(A) site cluster over the total number of reads for all clusters in the gene was calculated.

For 3′-seq [20], we used pre-processed “final” datasets for downstream analysis (see “Availability of data and materials” below). A similar approach was taken as above with a few modifications. Instead of using PolyAsite annotations, we determined the set of observed poly(A) site clusters by merging both brain and skeletal muscle datasets and scanned for clusters using an in-house Python script (find_sites.py, available on the QAPA GitHub page). The poly(A) sites were then quantified as above and similar PAU values were computed.

### Comparison between QAPA and 3′-end sequencing

For RNA-seq datasets, QAPA was applied using a human 3′ UTR library (hg19) as described above. We excluded genes with less than 100 nt between the 3′ ends of the proximal poly(A) site and the furthest downstream distal site.

For A-seq2 analysis, we mapped poly(A) site clusters to 3′ UTRs by finding the 3′ UTR whose 3′ end overlaps with the cluster. Next, we only considered 3′ UTRs expressed at least 5 TPM in both RNA-seq and A-seq2 in at least one of two replicates. We restricted our PPAU comparison to genes with exactly two 3′ UTRs. In some cases, there were poly(A) site clusters in A-seq2 that were not near a 3′ end of a 3′ UTR; in this case, we next added their TPMs to those of the 3′ UTRs whose 3′ end was first one downstream of the cluster. Total gene expression was measured by taking the sum of the TPMs of the two 3′ UTRs for that gene in that sample. We then computed the PPAU for each gene, in each sample, for each method. To ensure that we were comparing high confidence events, we removed genes whose PPAUs varied by more than 10% between replicates for a sample for both methods. We then computed the average PPAUs between replicates and used those for comparison. Replicates from each condition and method then were combined by taking the mean.

For analysis of differential 3′ UTR usage between RNA-seq and 3′-seq, we used a variable expression threshold rather than the fixed 5 TPM threshold used for A-seq2. First, we separately transformed the expression levels for each gene into a percentile between 10 to 90 (step size = 10) independently for each method. Next, at each percentile *p*, we considered the intersection of genes expressed above *p* in RNA-seq, and similarly for 3′-seq. We then required genes to have proximal 3′ UTR non-zero expression for both methods in the same tissue type. Within this intersection, the overlap of genes with APA changes between both methods was calculated where we require a |∆PPAU| > 10 between brain and skeletal muscle to define an APA change.

### Benchmarking of QAPA using simulated RNA-seq data

To evaluate QAPA against other RNA-seq-based methods for APA inference, we generated a synthetic RNA-seq dataset containing 200 mouse multi-3′ UTR genes with minimum 3′ UTR length of 100 nt across two conditions, each with three simulated biological replicates. For each gene, the proximal 3′ UTR isoform was assigned two PPAU values (one per condition). For the first condition, the PPAU is uniformly sampled from either a low usage range (10–49%) or high usage range (50–90%). For the second condition, the PPAU is uniformly sampled from the opposite range of the first condition along with an added restriction such that the minimum difference between the two conditions is at least 20%. The total PAU of all the distal isoforms was then set to 100% minus PPAU, and was allocated uniformly at random among the various distal isoforms if there was more than one. Through this sampling procedure, we generated 50 lengthening and 50 shortening events with |∆PPAU| > 20, as well as 100 non-changing events as a negative control (|∆PPAU| < 20). To simulate different coverage levels, baseline coverage for each gene was uniformly sampled between 10 to 50×. These parameters were then supplied to the R package polyester [71] to simulate paired-end 100-nt reads from the mouse genome (mm10), with Illumina error rate and GC bias models enabled (error_model = “illumina5”, gc_bias = 1).

We compared QAPA with three other methods: Roar v1.10.0 [26], DaPars v0.9.0 [25], and GETUTR v1.0.3 [27]. For each method, we provided annotations based on our QAPA 3′ UTR library to ensure that the same set of 3′ UTRs were interrogated. For Roar, the analysis was carried out using the supplied roarWrapper_multipleAPA.R script. Results were filtered for events with FDR < 0.1 and lengthening events were defined as having a roar value between 0 and 0.8, and shortening events with roar value > 1.2. For DaPars, the coverage cutoff was set to 10 and results were filtered for events with predicted proximal poly(A) sites that were within 100 nt of a QAPA-annotated proximal poly(A) site (FDR < 0.1). In DaPars, lengthening events were defined as those with Percentage of Distal Poly(A) Usage Index (PDUI) group difference (PDUI_Group_diff) < −0.2 and shortening events with PDUI_Group_diff > 0.2. For GETUTR, we used the default settings and results were filtered for predicted proximal poly(A) sites within 100 nt of a QAPA-annotated proximal poly(A) site. For GETUTR, the polyadenylation cleavage site (PCS) scores from the three replicates were averaged for each condition. Lengthening events were defined as having a change (∆) in PCS score > 0.2, while shortening events have a ∆PCS < −0.2. For analysis of human brain and skeletal RNA-seq datasets as shown in Additional file 1: Figure S3c, relaxed thresholds were applied to correspond with the RNA-seq versus 3′-seq analysis described above: roar: 0–0.9 and > 1.1 for lengthening and shortening, respectively: DaPars, −0.1 and 0.1, and GETUTR, 0.1 and −0.1.

To measure the run times of each method, we selected four representative samples from the Hubbard et al. [29] dataset: two replicates from DIV − 8 and two replicates from DIV 28. Each sample was randomly down-sampled to 20 million paired-end reads. Each method was then run twice on all four samples and the run times were averaged. For Roar, DaPars, and GETUTR, reads were first aligned to the mouse genome (mm10) using HISAT [72]. Where the methods used parallel computing, multiprocessing was enabled using eight threads. All computation was carried out on a cluster equipped with four Intel Xeon E7–4830 2.13 Ghz 8-core processors, 256 GB RAM, and running CentOS Linux 7 (x86–64) operating system.

### APA analysis of neuronal differentiation

#### Pre-processing

QAPA was applied using a mouse 3′ UTR library (mm10). We kept 3′ UTRs that had a total gene expression of at least 3 TPM in at least 29/31 samples across all stages and replicates. In order to avoid overlapping non-strand specific RNA-seq reads due to two genes converging into each other, we excluded gene pairs whose distal 3′ UTRs had 3′ ends that were within 500 nt of each other on the genome. We also excluded genes with aUTR lengths of less than 100 nt to reduce potentially noisy estimates due to small differences in length between proximal and distal 3′ UTR sequences. We defined the change in proximal poly(A) site usage (∆PPAU) as the difference between the median PPAU of ESC group (DIV −8 and −4) replicates and the median PPAU of the neuron group (DIV 7, 16, 21, and 27) replicates.

#### Principal component analysis

To extract patterns of APA during ND, principal component analysis (PCA) was performed on mean-centered PPAU values using the R function prcomp().

#### Gene set enrichment analysis

We applied gene set enrichment analysis (GSEA) [37] on gene lists containing either lengthening 3′ UTRs or shortening ones. GSEA analysis requires a real-valued score for each gene in each list in each phenotype. For this score, we used the PPAU values and assigned a binary phenotype for each sample that indicated whether the sample was in the ESC group (as defined above) or the NEURON group. We tested the enrichment of gene sets contained in the GMT file: “MOUSE_GO_bp_no_GO_iea_symbol.gmt”. These are mouse-specific Enrichment Map Gene Sets downloaded from http://baderlab.org/GeneSets [73]. GSEA was performed from command line with the options: collapse = false, mode = Max_probe, norm = meandiv, nperm = 1000, permute = phenotype, metric = Ratio_of_Classes, set_max = 300, set_min = 20, include_only_symbols = true, make_sets = true, median = false. Only the gene list associated with the lengthening 3′ UTRs had any significantly enriched terms.

Significant terms were summarized using Enrichment Map [73] in Cytoscape [74] with settings: *p* value cutoff = 0.01, FDR Q-value cutoff = 0.025, overlap coefficient = 0.9. Clusters of related terms in the network were manually summarized by extracting common keywords using the WordCloud plugin (http://baderlab.org/WordCloud).

#### Differential gene expression analysis

DESeq2 [40] was used to compare gene expression changes between ESC samples (DIV −8 and −4) as one condition versus mature neuronal samples (DIV 7, 16, 21, and 28) as the contrasting condition. We defined differentially expressed genes as those with a |log_2_ fold change| > 1.5 and FDR < 0.01, where fold change is defined as the expression in neural samples divided by the expression in ESC samples.

DEXSeq [41] was used to compare 3′ UTR isoform expression changes between ESC and mature neurons. As per the method’s procedure, 3′ UTR isoforms were collapsed and segmented into adjacent bins demarcated by each isoform’s boundaries. In particular, we denote the 5′-most bin in the 3′ UTR as the proximal bin, which is associated with the “common UTR regions” (cUTR) — the region common to proximal and distal isoforms. We denote the remaining bin(s) located 3′ to the proximal bin as distal bin(s), which are associated with “alternative UTR regions” (aUTRs) originating from one or more distal isoforms. We defined a bin to be significantly differentially expressed if it had a |log_2_ fold change| > 0.5 and FDR < 0.1. For the latter, the same FDR was used as by the DEXSeq authors. In the case of multiple distal 3′ UTRs, we required a significant change for at least one of the distal bins. We then classified each 3′ UTR lengthening event into three classes. First, a *Switch* event is defined by a significant increase in a distal bin usage and unchanged or decrease (i.e., log_2_ fold change < 0.5) in proximal bin usage reflecting reciprocal changes in expression between proximal and distal isoforms. A *Long-Up* event is defined by a significant increase in both proximal and distal bin usage. A *Short-Down* event is defined by a significant decrease in proximal bin usage and non-significant change in distal bin usage.

#### Differential alternative splicing analysis

Alternative splicing analysis was carried out using vast-tools v0.1.0 [50, 75] (default settings). Splicing events that were differentially regulated between ESCs and neurons were identified using the *vast-tools diff* module (--minReads = 20).

#### Transcription initiation sites analysis

To identify transcription initiation sites, whole transcript abundances were measured using Sailfish [28] on GENCODE [33] basic gene annotation (version M9). Transcripts with the same distinct transcription initiation sites were aggregated by calculating the maximum expression across all samples. Expressed initiation sites were defined as having at least 3 TPM.

### Features used in the APA model

#### Dinucleotide content (32 real-valued features)

There were 32 dinucleotide content features per poly(A) site. Among these, 16 were the dinucleotide frequencies in the 300 nt upstream of the poly(A) site. The other 16 were the frequencies of each in the downstream 300 nt.

#### Poly(A) signals and enhancer elements (19 binary features)

A total of 18 poly(A) signal features were compiled from [13, 14]: AAUAAA, AAGAAA, AAUACA, AAUAGA, AAUAUA, AAUGAA, ACUAAA, AGUAAA, AUUAAA, CAUAAA, GAUAAA, UAUAAA, AAUAAU, AACAAA, AUUACA, AUUAUA, AACAAG, AAUAAG. Each signal was represented as a binary feature indicating whether or not it is present in the 50 nt upstream of the poly(A) site. In addition, there was one binary feature indicating whether or not the upstream enhancer element UGUA was present in the 50 to 100 nt upstream of the poly(A) site.

#### Poly(A) site dinucleotide (16 binary features)

The dinucleotide at a poly(A) site is recorded by taking the 2-mer sequence at position (*t* – 1, *t*) where *t* is the 3′ coordinate of the poly(A) site. This dinucleotide was represented using a one-hot encoding.

#### RNA-binding protein motifs and secondary structure accessibility (1218 real-valued features)

A total of 203 IUPAC motifs from RNAcompete were scanned upstream and downstream of each poly(A) site [53]. To account for the accessibility of the observed motif in each 3′ UTR, we scored each motif target site based on the probability of the site forming a local secondary structure. To do this, RNAplfold [76] was used to compute local RNA secondary structures over small windows of a given size (W = 200, L = 150, U = 1; as per Li et al. [54]). This produces position-specific probabilities that a base is unpaired. For each target site, an accessibility score was calculated by taking the average of all unpaired probabilities. Finally, for each motif, the accessibility scores are aggregated into six 100-nt discrete bins with respect to the poly(A) site (denoted as position = 0): (−300, −200), (−200, −100), (−100, 0), (0, 100), (100, 200), and (200, 300). Motif hits that spanned bin boundaries (e.g., starting at −102 and finishing at −98) were counted in both bins. Scores within each bin are summed, giving the expected number of accessible target sites within each bin.

#### Conservation (four real-valued features)

Sequence conservation from the PhyloP 60-way track [77] for the mouse genome (mm10) was downloaded from the UCSC Genome Browser. For each poly(A) site, conservation scores were extracted using bedtools intersect [68] and summarized by taking the average within 100-nt bins in the region 200 nt downstream and 200 nt upstream of the poly(A) site. In other words, we used the following bins: (−200, −100), (−100, 0), (0, 100), (100, 200).

### Feature selection

We carried out a preliminary feature selection step using the R package caret to eliminate non-informative features. In particular, we removed features that had zero variance using the function nearZeroVar(). We also used the function findCorrelation() to identify highly correlated pairwise features (Pearson correlation R ≥ 0.8). If two features are highly correlated, then the feature with largest mean absolute correlation with other features was removed.

### Model training and evaluation

We kept a random 80% of the data for training and held out the remaining 20% for testing. We used stratified sampling to maintain the relative balance of proximal and constitutive 3′ UTR events in the training and test sets. To train the regression model, we evaluated a number of different machine learning algorithms that are available as R packages: linear regression with LASSO regularization using glmnet [78], random forests using randomForest [79], gradient tree boosting using xgboost [80]. For each method, we used the R package caret to select the optimal hyperparameters—it performs a method-specific grid search over different hyperparameter settings. Each parameterized model was tested by tenfold cross-validation (CV). The same seed was used when training each method to ensure that the same fold samples were used during CV in order to remove inter-method variability in the test error statistics due to different training sets. For each method, the best CV model was selected based on having the lowest root mean squared error (RMSE):

$$ RMSE=\sqrt{\frac{1}{n}\sum \limits_{i=1}^n{\left({\widehat{y}}_i-{y}_i\right)}^2} $$

where ${\widehat{y}}_i$ is the predicted value and *y*_*i*_ is the observed value for data point *i*. The final model was then trained on the entire training dataset using the parameters from the best CV model. Each model was then applied to the held-out test dataset to assess relative performance.

The parameters selected by caret’s CV for each method are as follows:

- *glmnet*: alpha = 1, lambda = 0.2858073
- *randomForest*: ntree = 500, mtry = 330
- *xgboost*: nrounds = 50, max_depth = 3, eta = 0.3, gamma = 0, colsample_bytree = 0.8, min_child_weight = 1, subsample = 1

To measure variable importance in random forests, as shown in Fig. 6b, c, the R function importance() from the randomForest package was used. Briefly, each training example was evaluated on the same random forests model that it was trained on; but only on decision trees where the example was not used during training. These trees are known as out-of-bag (OOB) trees. For each OOB tree, a prediction is made on each example and the mean squared error is computed. Next, each feature variable is permuted and evaluated on the tree. The difference in mean-squared error between the observed data and permuted data is recorded. Finally, the average difference for each variable over all trees is computed, normalized by the standard error.

## Additional files

Additional file 1: Supplementary figures. (PDF 4990 kb)

Additional file 2: Neuronal differentiation APA. This tab-delimited file contains the QAPA-estimated PAU and Sailfish TPM values of each 3′ UTR isoform for samples from the Hubbard et al. [29] RNA-seq dataset. (TXT 6281 kb)

Additional file 3: Poly(A) site feature matrix for modeling the APA regulatory code. This tab-delimited file contains feature matrix used to build a regression model of poly(A) site strength. Each row is a training/test example of a poly(A) site based on proximal and constitutive 3′ UTRs. Except for the first six columns, each column is a feature vector describing a particular property of the example’s poly(A) site. The response vector (“y”) is the average PAU of all samples from the Hubbard et al. RNA-seq dataset. Polyadenylation signal features begin with the prefix “PAS”. Poly(A) site dinucleotide features have the suffix “_pasite”. Overall upstream and downstream dinucleotide contents have the suffices “_upcontent” and “_downcontent”, respectively. Conservation features have the prefix “phyloP60way” followed by the bin location. Cis RBP motif features have the prefix “cis”. (TXT 18071 kb)

## Abbreviations

APA: Alternative polyadenylation
AS: Alternative splicing
AUC: Area under the receiver operating characteristic curve
DIV: Days *in vitro*
ESC: Embryonic stem cells
GO: Gene Ontology
GSEA: Gene set enrichment analysis
mRNA: Messenger RNA
ND: Neuronal differentiation
PAU: Poly(A) site usage
PCA: Principal component analysis
PPAU: Proximal poly(A) site usage
RBP: RNA-binding protein
RMSE: Root mean squared error
TPM: Transcripts per million
UTR: Untranslated region
