# Supplementary material for: QAPA: a new method for the systematic analysis of alternative polyadenylation from RNA-seq data
Source: Genome Biol. 2018 Mar 28;19:45. doi: 10.1186/s13059-018-1414-4 (PMC5874996; doi:10.1186/s13059-018-1414-4)
Supplement: Supplementary file 1 — Supplementary figures. (PDF 4990 kb) [file 13059_2018_1414_MOESM1_ESM.pdf]

Supplementary Figures

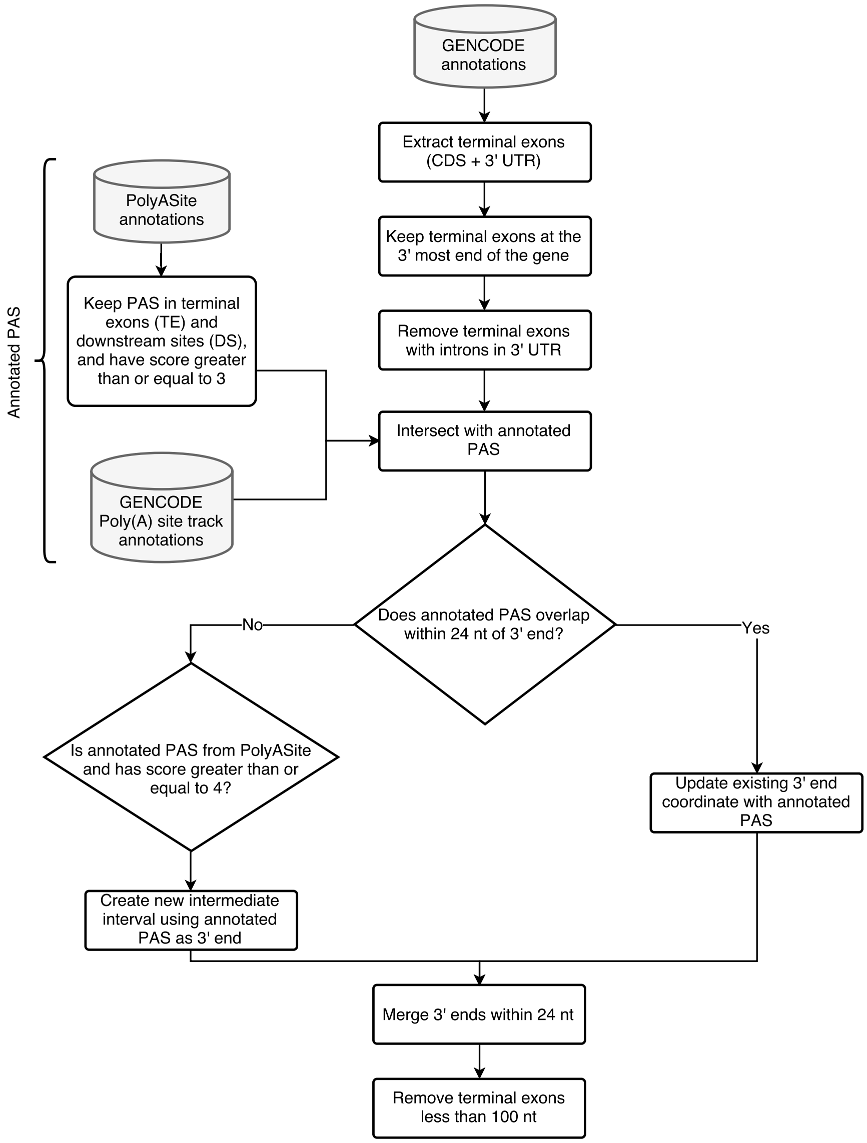

Figure S1: **Flowchart outling the QAPA 3′ UTR extraction procedure.** Terminal exon genomic intervals are extracted from GENCODE gene model annotations [1]. After several filtering steps, the intervals are annotated with additional PAS annotation sources: GENCODE Poly(A) site track [1] and PolyASite [2]. If an interval's 3′ end overlaps within 24 nt of an annotated PAS, then the 3′ end is updated to that of the annotated PAS. Otherwise, a new 3′ interval is created. Finally, the resulting interval list is processed such that intervals with 3′ ends within 24 nt are merged, followed by removal of sequences shorter than 100 nt.

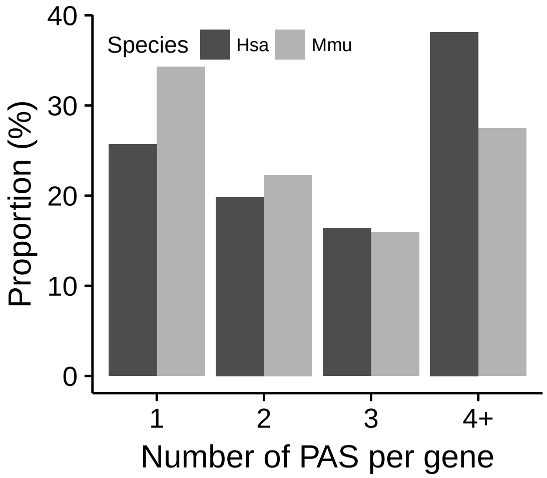

Figure S2: **APA is widespread in human and mouse**. Barplot showing the proportion of genes $N = \{1, 2, 3, 4+\}$ PASes in human and mouse from QAPA-annotated $3'$ UTR libraries.

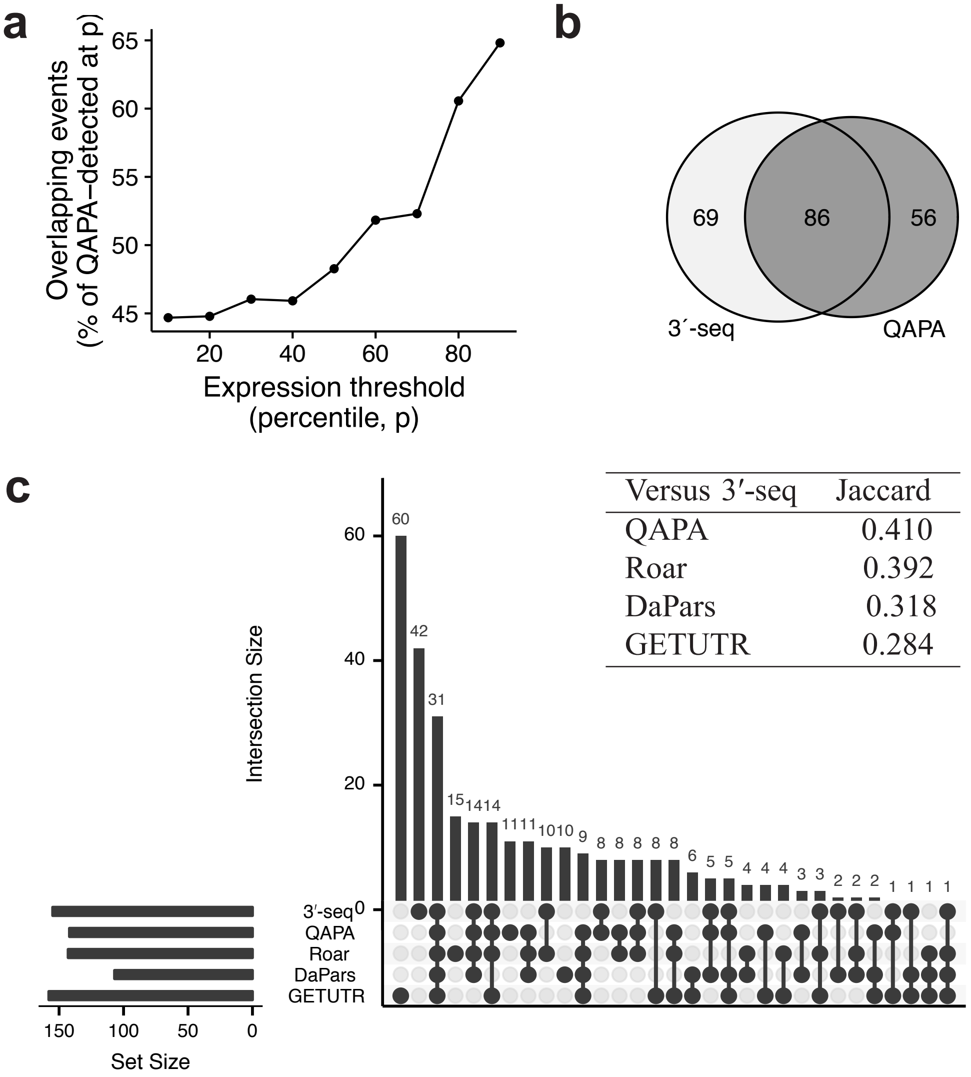

Figure S3: **Comparison of APA events between human brain and skeletal muscle tissues detected by QAPA and 3′-seq.** (a) The overlap of APA events detected by QAPA and 3′-seq [3] with $|\Delta PPAU| > 10$ compared at various minimum gene expression thresholds. Gene expression was segmented at percentiles between 10% and 90% with step size of 10. The proportion of QAPA-detected APA events that are common with 3′-seq at a specific percentile is shown (y-axis). (b) Venn diagram showing the overlap of APA events detected by QAPA and 3′-seq at $p = 0.8$. (c) UpSet plot [7] summarizing the overlap of APA events detected by 3′-seq, QAPA and three other methods: Roar [4], DaPars [5], and GETUTR [6]. Each vertical bar (y-axis) indicates the number of overlapping genes between the methods indicated by the dot(s) below. The top-right table summarizes the pairwise overlap between each method and 3′-seq measured using the Jaccard index, defined as $J(A,B) = \frac{|A \cap B|}{|A \cup B|}$, where A and B are sets of events. The same filtering criteria from (A) and (B) were used.

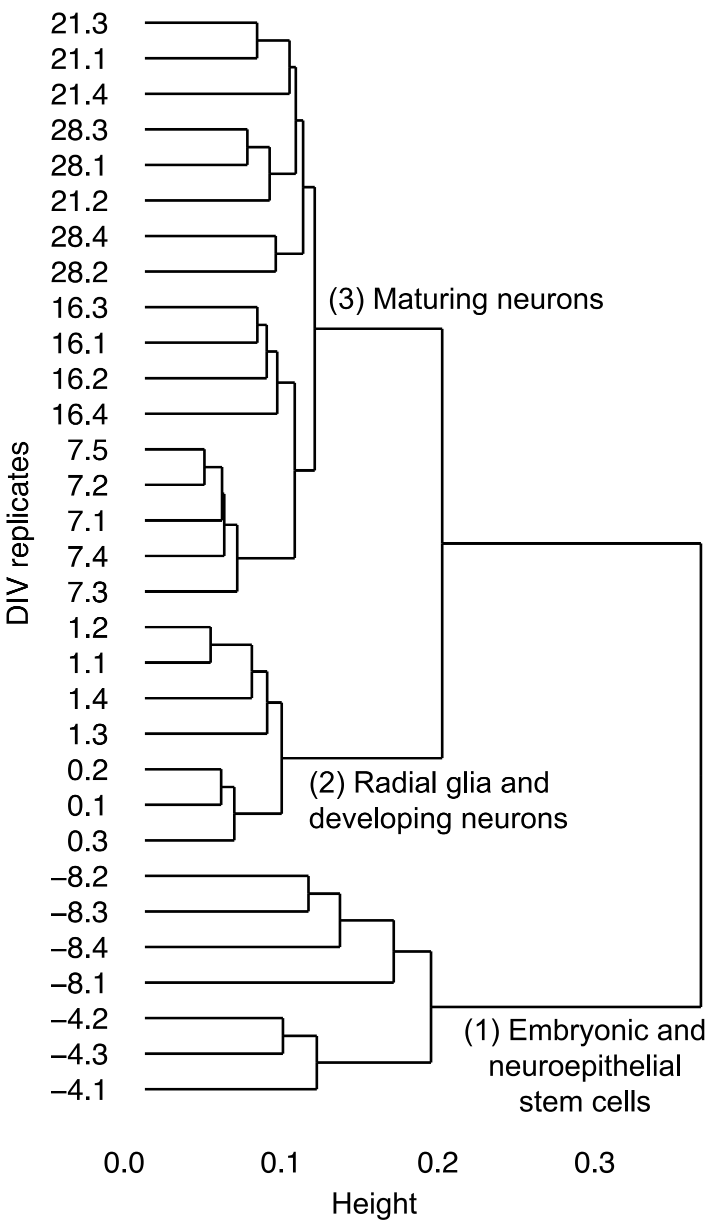

Figure S4: **Hierarchical clustering of replicates from Hubbard et al. [8] RNA-seq.** Dendrogram showing the clustering of PAU values of Hubbard et al. [8] RNA-seq samples, showing that replicates cluster together, and more broadly, samples from related developmental stages cluster together.

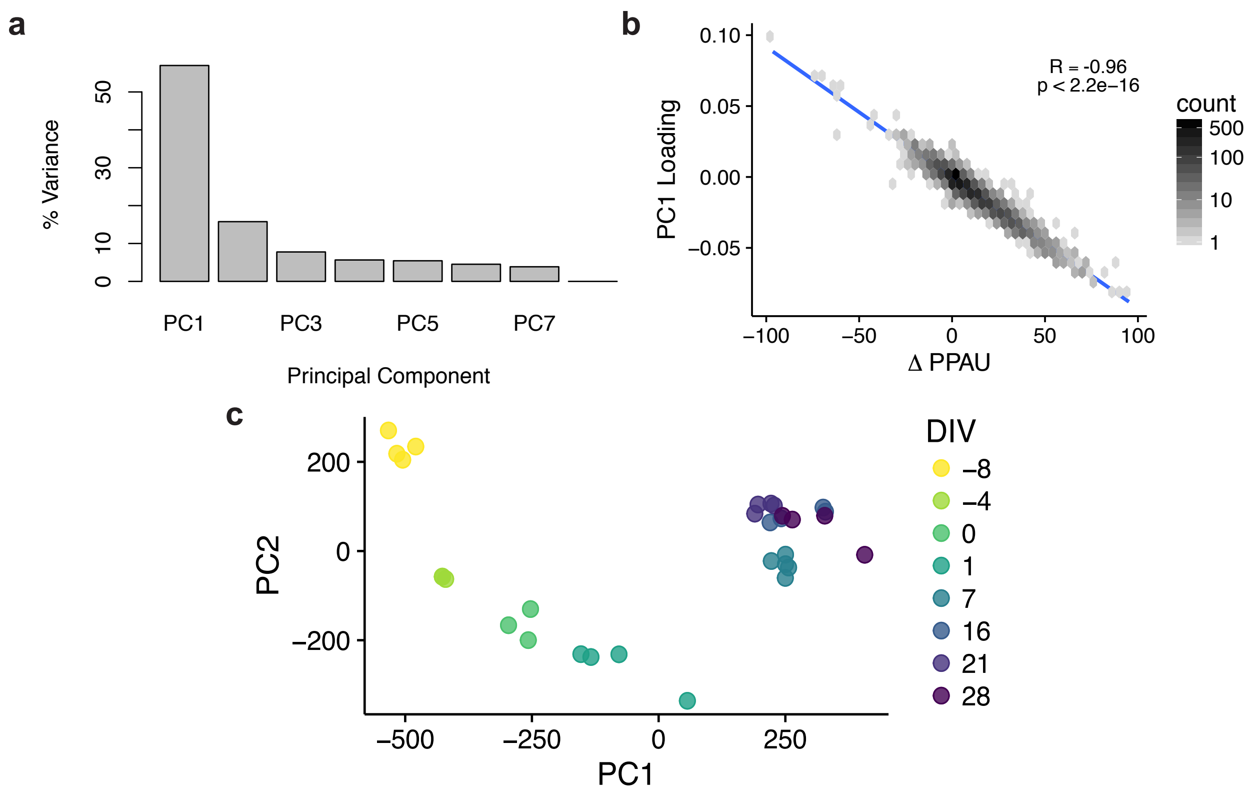

Figure S5: **Principal Component Analysis (PCA) of PPAU values on a neuronal differentiation time-series RNA-seq [8] RNA-seq.** (a) barplot showing the % variance explained for each Prinicpal Component (PC). (b) To verify that PC1 corresponds with time, the computed PC1 loadings assigned to each 3′ UTR is compared to change in PPAU ($\Delta PPAU$) between the ESC stages (DIV -8 and -4) and neuron stages (DIV 7-28). Each stage is summarized by taking the median PPAU of each replicate. The line of best fit is indicated by the blue line. (c) PCA was repeated but performed on replicates individually. Each coloured dot represents a replicate.

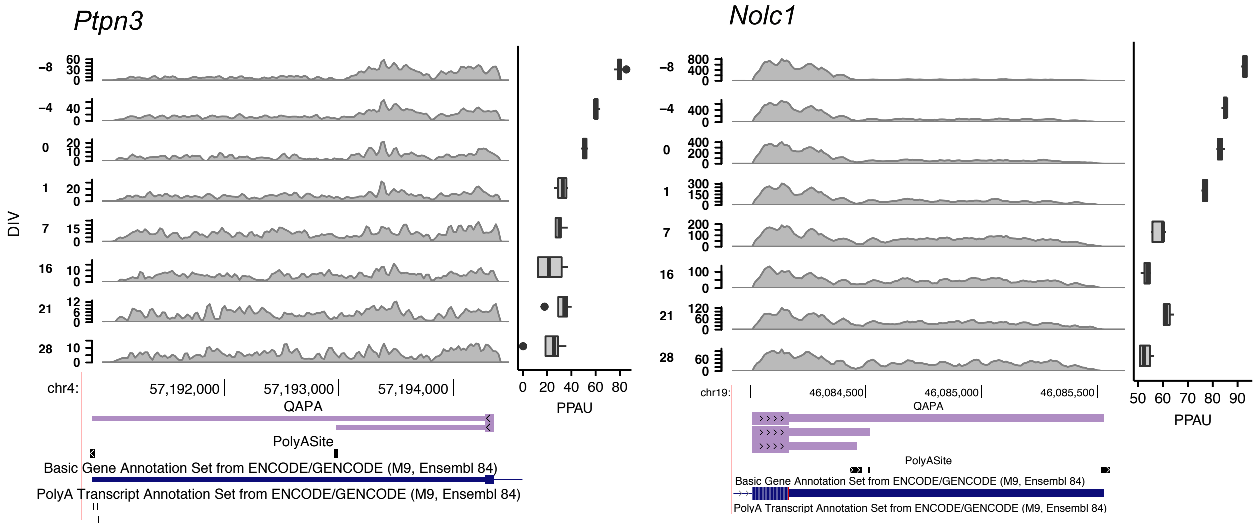

Figure S6: **Examples of 3′ UTR lengthening during neuronal differentiation.** RNA-seq read coverage plots for two 3′ UTR lengthening examples. Below each coverage plot is a UCSC Genome Browser schematic of each 3′ UTR. Four annotation tracks are shown. From top to bottom, QAPA-annotated 3′ UTR models, PolyASite [2] with score >= 3, GENCODE [1] gene annotation models, and GENCODE Poly(A) annotations. The boxplot to the right show the PPAU values from replicates in each corresponding DIV stage to the left. *Ptpn3* is shown in the reverse strand orientation.

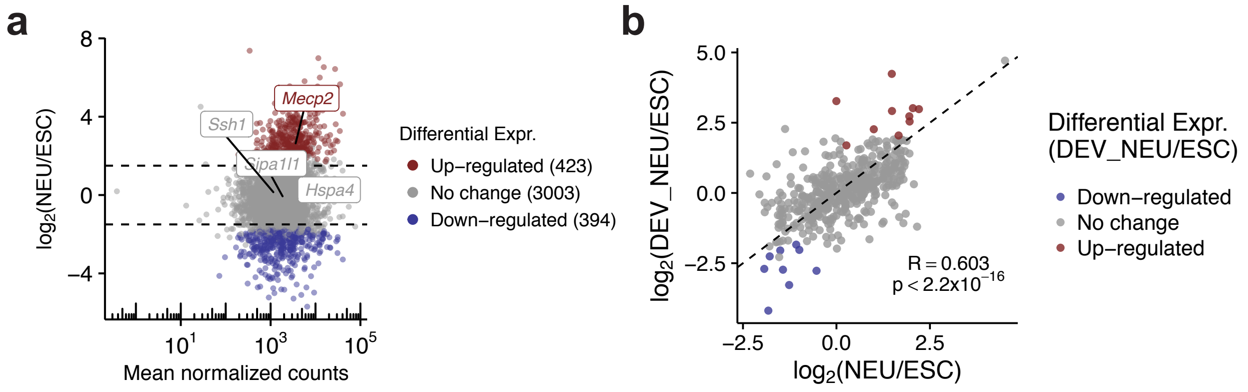

Figure S7: **Most 3′ UTR lengthening and shortening genes do not change in steady-state gene expression during neuronal differentiation.** (A) MA plot from DESeq2 [9] differential gene expression analysis between mature neurons (NEU) and ESCs. The x-axis indicates the mean normalized read count and the y-axis indicates the $\log_2$-fold change between NEU and ESC. Genes with statistically significant increased and decreased expression are indicated by red and blue dots, respectively [$|\log_2$ fold change— > 1.5, $FDR < 0.01$]. The dashed horizontal lines indicate the $\log_2$ fold change thresholds. (B) DESeq2 was repeated to compare ESCs with an earlier neuronal stage (DIV 1). The $\log_2$ fold change of the 460 genes with inferred APA but no differential expression changes in the first comparison was compared to the second: ESCs versus mature neurons (NEU; x-axis), and ESCs versus DEV_NEU (y-axis). Dots are coloured according to differential expression status of the DEV_NEU/ESC comparison. The dashed line represents the reference diagonal.

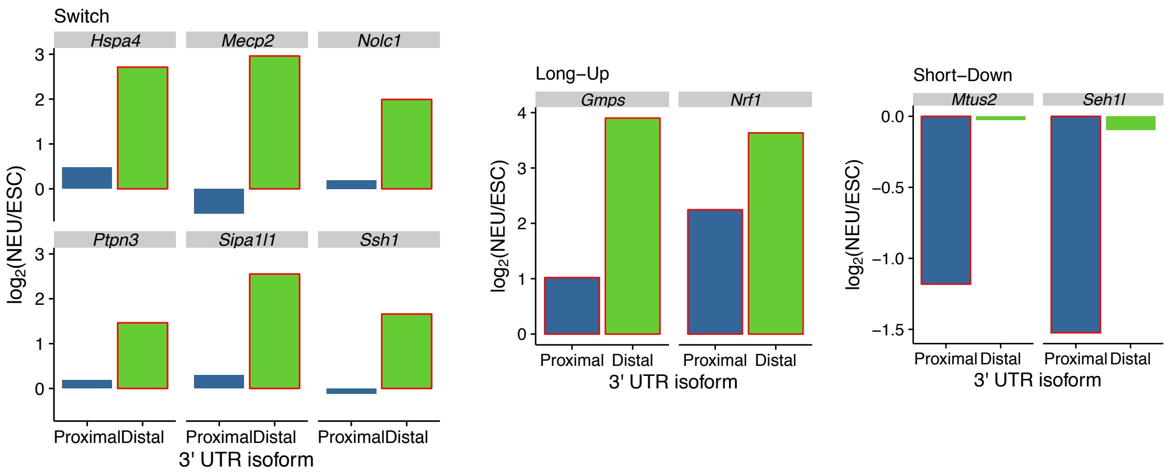

Figure S8: **3′ UTR lengthening arises from isoform expression changes.** DEXSeq [10] was used to compare isoform expression changes of proximal and distal isoforms of genes with APA. Genes were then classified into three classes: *Switch*, *Long-Up*, and *Short-Down*. Examples of genes and the corresponding $\log_2$ fold changes of proximal (blue) and distal (green) isoforms are shown. Bars with a red outline indicate a statistically significant change ($\log_2$ fold change > 1 and FDR $<$ 0.10).

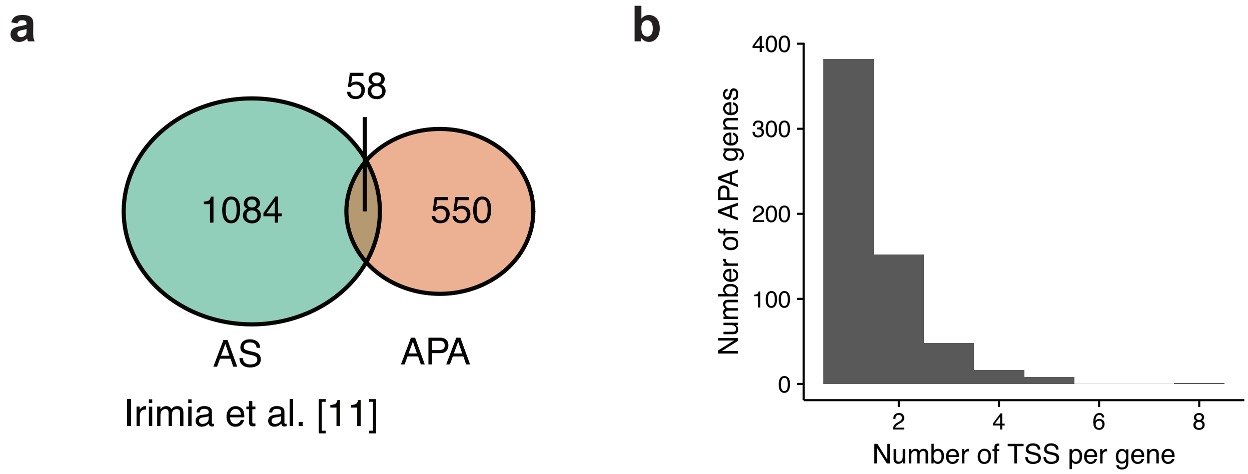

Figure S9: **Overlap of 3′ UTR lengthening and shortening genes with differential alternative splicing (AS) and transcription start sites.** (a) Venn diagram showing the overlap between QAPA-inferred APA genes and genes with previously identified neural-regulated AS events from independent RNA-seq sources [11]. (b) Histogram showing the number of distinct transcription start sites per gene among the 608 QAPA-inferred APA genes.

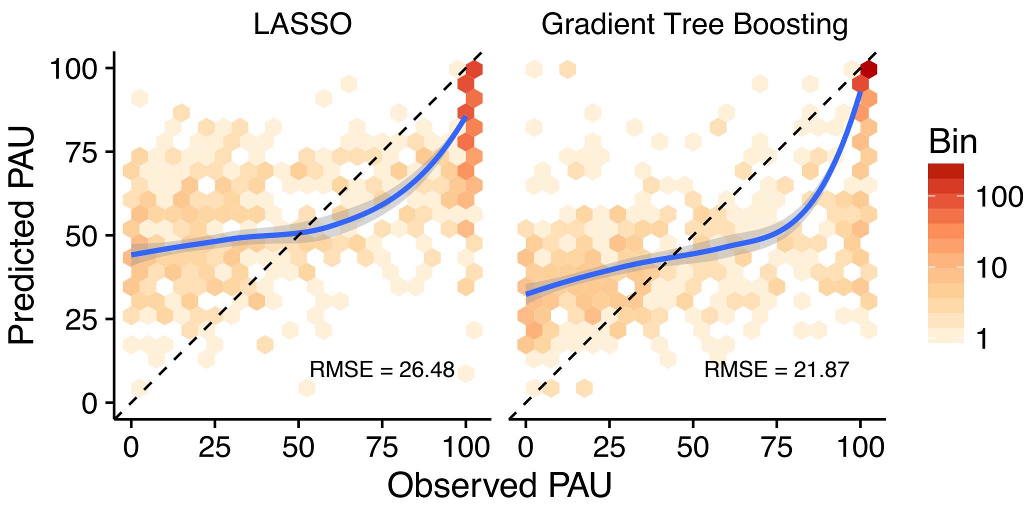

Figure S10: **Test set performance of LASSO and gradient boosted trees.** Hexbin scatterplots showing the observed PPAU with the predicted PPAU for each model. Performance was evaluated by computing the root mean squared error (RMSE). Bins are coloured by number of data pointsand dashed line indicates the reference diagonal. The blue line represents polynomial spline of best fit on the data.

References

[1] Harrow J, Frankish A, Gonzalez JM, Tapanari E, Diekhans M, Kokocinski F, et al. GENCODE: The reference human genome annotation for the ENCODE project. Genome Res. 2012;22(9):1760–1774.

[2] Gruber AJ, Schmidt R, Gruber AR, Martin G, Ghosh S, Belmadani M, et al. A comprehensive analysis of 3' end sequencing data sets reveals novel polyadenylation signals and the repressive role of heterogeneous ribonucleoprotein C on cleavage and polyadenylation. Genome Res. 2016 aug;26(8):1145–1159.

[3] Lianoglou S, Garg V, Yang JL, Leslie CS, Mayr C. Ubiquitously transcribed genes use alternative polyadenylation to achieve tissue-specific expression. Genes Dev. 2013 nov;27(21):2380–96.

[4] Grassi E, Mariella E, Lembo A, Molineris I, Provero P. Roar: detecting alternative polyadenylation with standard mRNA sequencing libraries. BMC Bioinformatics. 2016;17(1):423.

[5] Xia Z, Donehower La, Cooper Ta, Neilson JR, Wheeler Da, Wagner EJ, et al. Dynamic analyses of alternative polyadenylation from RNA-seq reveal a 3'-UTR landscape across seven tumour types. Nat Commun. 2014 jan;5:5274.

[6] Kim M, You BH, Nam JW. Global estimation of the 3' untranslated region landscape using RNA sequencing. Methods. 2015 jul;83:111–7.

[7] Conway JR, Lex A, Gehlenborg N. UpSetR: an R package for the visualization of intersecting sets and their properties. Bioinformatics. 2017 sep;33(18):2938–2940.

[8] Hubbard KS, Gut IM, Lyman ME, McNutt PM. Longitudinal RNA sequencing of the deep transcriptome during neurogenesis of cortical glutamatergic neurons from murine ESCs. F1000Research. 2013 jan;2:35.

[9] Love MI, Huber W, Anders S. Moderated estimation of fold change and dispersion for RNA-seq data with DESeq2. Genome Biol. 2014;15(12):550.

[10] Anders S, Reyes A, Huber W. Detecting differential usage of exons from RNA-seq data. Genome Res. 2012 jun;22(10):gr.133744.111–.

[11] Irimia M, Weatheritt RJ, Ellis JD, Parikshak NN, Gonatopoulos-Pournatzis T, Babor M, et al. A Highly Conserved Program of Neuronal Microexons Is Misregulated in Autistic Brains. Cell. 2014 dec;159(7):1511–1523.
